# A Case Series of Metastatic Metaplastic Breast Carcinoma Treated With Anti-PD-1 Therapy

**DOI:** 10.3389/fonc.2021.635237

**Published:** 2021-06-08

**Authors:** Isaac Kim, Venkatesh Rajamanickam, Brady Bernard, Brie Chun, Yaping Wu, Maritza Martel, Zhaoyu Sun, William L. Redmond, Katherine Sanchez, Reva Basho, Heather McArthur, David B. Page

**Affiliations:** ^1^ Earle A. Chiles Research Institute, Providence Cancer Institute, Portland, OR, United States; ^2^ Department of Medicine, Cedars Sinai Medical Center, Los Angeles, CA, United States

**Keywords:** metaplastic breast cancer, TNBC, immunotherapy, PD-L1, PI3K

## Abstract

Metaplastic breast cancer is a rare and often chemo-refractory subtype of breast cancer with poor prognosis and limited treatment options. Recent studies have reported overexpression of programmed death ligand 1 (PD-L1) in metaplastic breast cancers, and there are several reports of anti-PD-1/L1 being potentially active in this disease. In this case series, we present 5 patients with metastatic metaplastic breast cancer treated with anti-PD-1-based therapy at a single center, with 3 of 5 cases demonstrating a response to therapy, and one of the responding cases being a metaplastic lobular carcinoma with low-level hormone receptor expression. Cases were evaluated for PD-L1 expression, tumor infiltrating lymphocytes (TILs), DNA mutations, RNA sequencing, and T-cell receptor sequencing. Duration of the response in these cases was limited, in contrast to the more durable responses noted in other recently published reports.

## Introduction

Metaplastic breast cancer (MBC) is a rare and aggressive subtype of breast cancer, comprising approximately 1% of all breast cancers, and is defined histologically as tumors that have epithelial differentiation into squamous and/or mesenchymal components, with multiple components often co-existing in the same tumor (1, 2). The current WHO classification of breast tumors further divides metaplastic carcinoma into additional subtypes: low grade adenosquamous, fibromatosis-like metaplastic, squamous cell, spindle cell, metaplastic with mesenchymal differentiation (including chondroid, osseous, or other types), mixed metaplastic, and myoepithelial carcinomas (3). There is limited understanding of the prognostic implications of various subtypes, and therefore are all clinically treated as a single entity (4). MBCs tend to present with a larger size, less frequent axillary nodal involvement, and have a higher rate of developing distant metastasis compared to other breast cancers (5, 6). They are frequently negative for estrogen receptor (ER), progesterone receptor (PR), and Human epidermal growth factor 2 (HER2) overexpression, with 85-89% of cases noted to be triple negative in recent analyses (6–9). However, compared to other triple negative breast cancers (TNBC), MBCs tend to have worse outcomes across all clinical stages, with 3-year overall survival for stage IV disease of 15% vs 22% for TNBC, and 64% for all other breast cancer types in one recent analysis of the National Cancer Database (10). MBCs also have poor response rates to cytotoxic chemotherapy compared to other types of breast cancer (5, 11, 12). As a result, there has been interest in evaluating novel strategies, including targeted therapies and immunotherapy (12, 13). The potential utility of immunotherapy for this disease has been highlighted by recent reports of metastatic MBC with durable responses to immune checkpoint blockade (14–16). Here, we present a case series of 5 patients with metastatic MBC treated with anti-PD-1 therapy.

## Materials and Methods

### Patients

**Figure 1:**
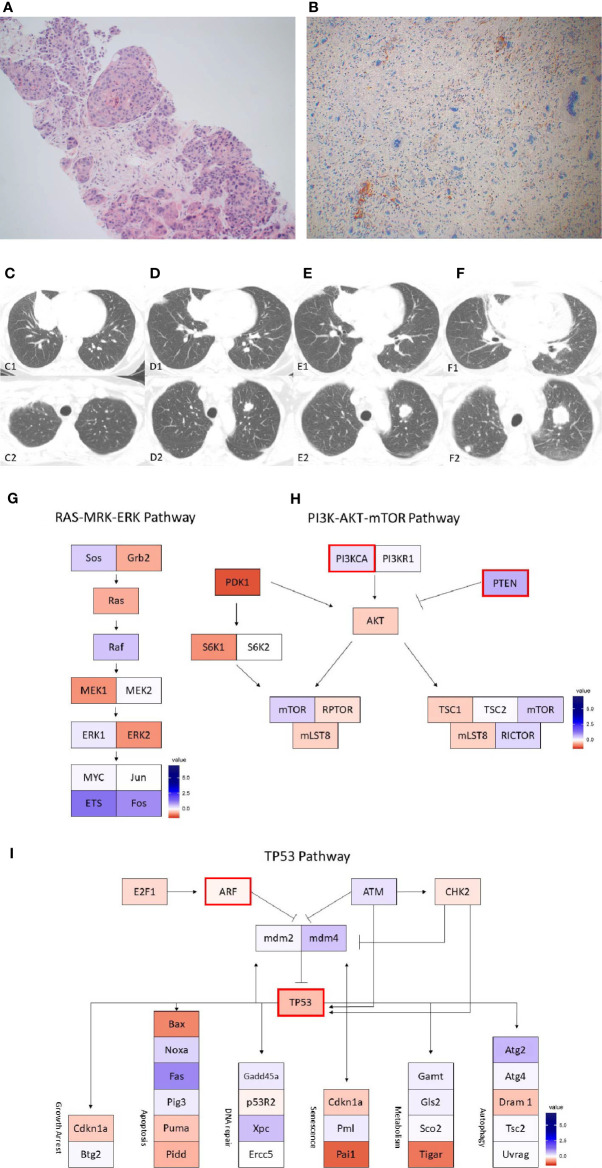
Case 1 **(A)** H&E image, showing metaplastic carcinoma with chondroid differentiation **(B)** PD-L1 by the Ventana PD-L1 SP263 assay **(C–F)**. Radiographic changes in Case 1 from **(C)** week 0, **(D)** 12 weeks, **(E)** 16 weeks, and **(F)** 24 weeks. Images C1 to F1 showing regression of the dominant right lung mass, then regrowth. Images C2 to F2 showing growth of an initially non-target left lung nodule. Images **(G–I)** show RNA expression heatmaps with modified z-scores of expression *vs.* non-metaplastic TNBC cases in pathways of interest for metaplastic breast cancer **(G)** RAS-MEK-ERK, **(H)** PI3K-AKT-mTOR **(I)** TP53. Genes with DNA mutations are outlined in red.

**Figure 2:**
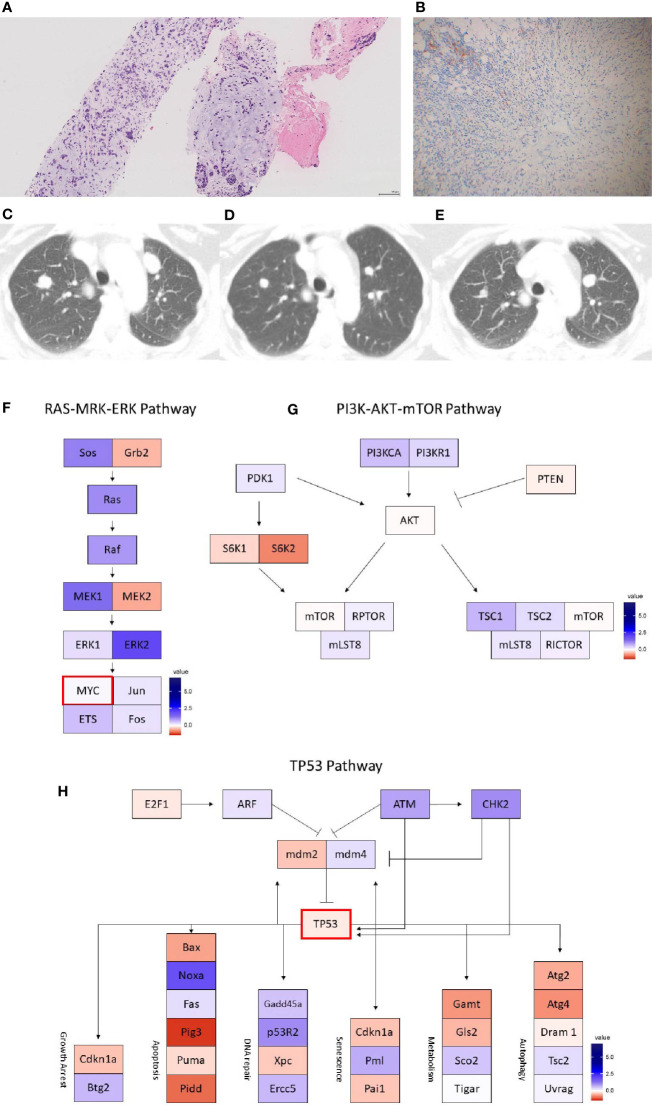
Case 2 **(A)** H&E image, showing metaplastic squamous carcinoma **(B)** PD-L1 by the Ventana PD-L1 SP263 assay **(C–E)**. Radiographic changes from **(C)** week 0, **(D)** 12 weeks, and **(E)** 24 weeks. A mixed, but overall partial response by RECIST criteria is noted initially **(D)** followed by progression **(E)**. Images **(F–H)** show RNA expression heatmaps with modified z-scores of expression *vs.* non-metaplastic TNBC cases in pathways of interest for metaplastic breast cancer **(F)** RAS-MEK-ERK, **(G)** PI3K-AKT-mTOR **(H)** TP53. Genes with DNA mutations are outlined in red.

**Figure 3:**
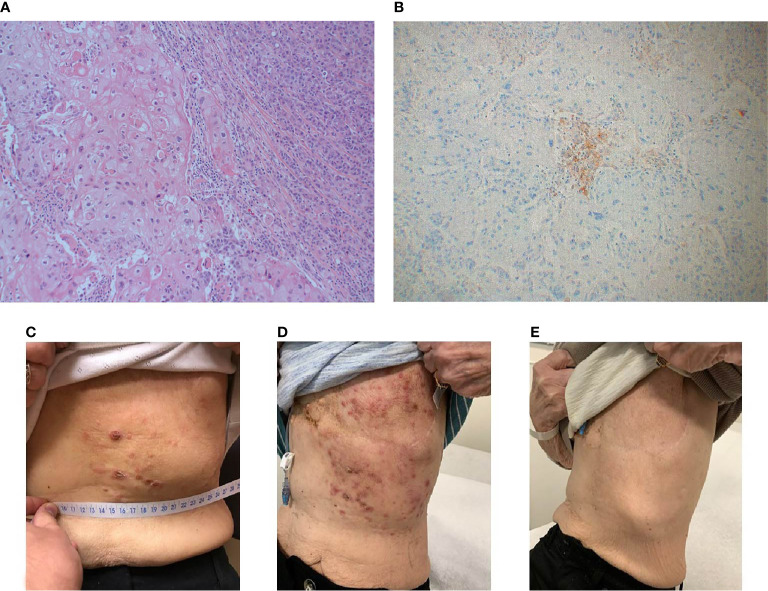
Case 3 **(A)** H&E image, showing mixed metaplastic squamous carcinoma and pleomorphic invasive lobular carcinoma **(B)** PD-L1 by the Ventana PD-L1 SP263 assay **(C–E)**. Lesions at baseline **(C)** initially appeared worsened at 4 weeks **(D)**, then demonstrated a complete clinical response by week 14 **(E)**.

**Figure 4:**
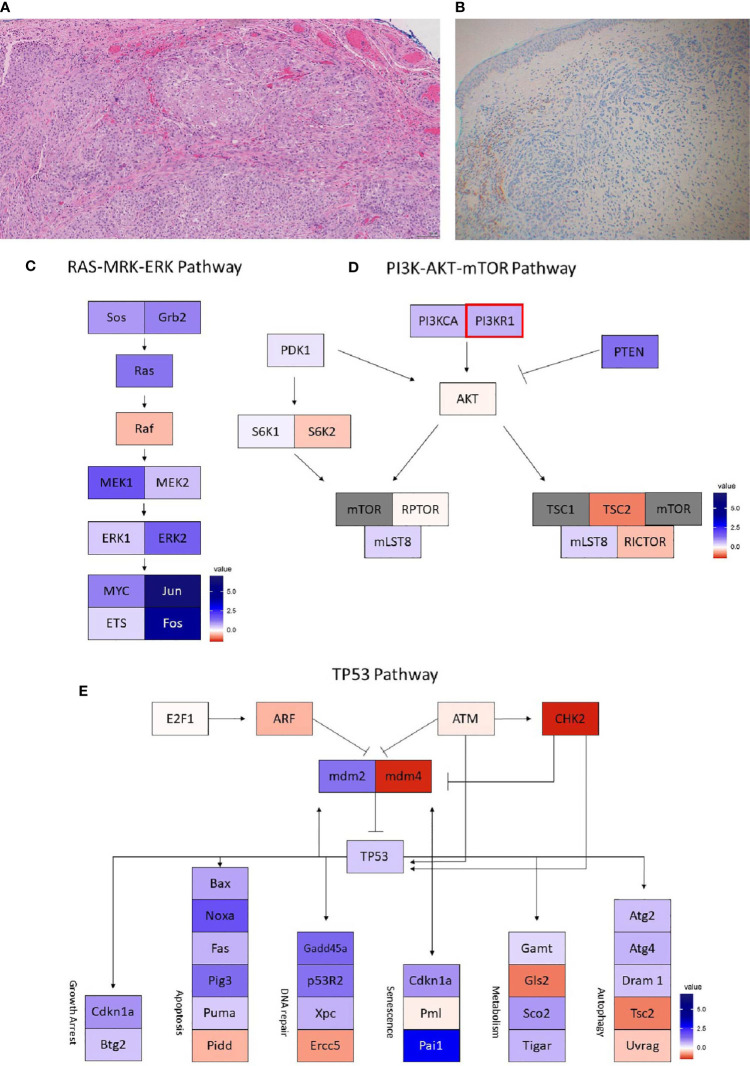
Case 4 **(A)** H&E image, showing metaplastic squamous carcinoma **(B)** PD-L1 by the Ventana PD-L1 SP263 assay. Images **(C–E)** show RNA expression heatmaps with modified z-scores of expression vs. non-metaplastic TNBC cases in pathways of interest for metaplastic breast cancer **(C)** RAS-MEK-ERK, **(D)** PI3K-AKT-mTOR **(E)** TP53. Genes with DNA mutations are outlined in red.

**Figure 5:**
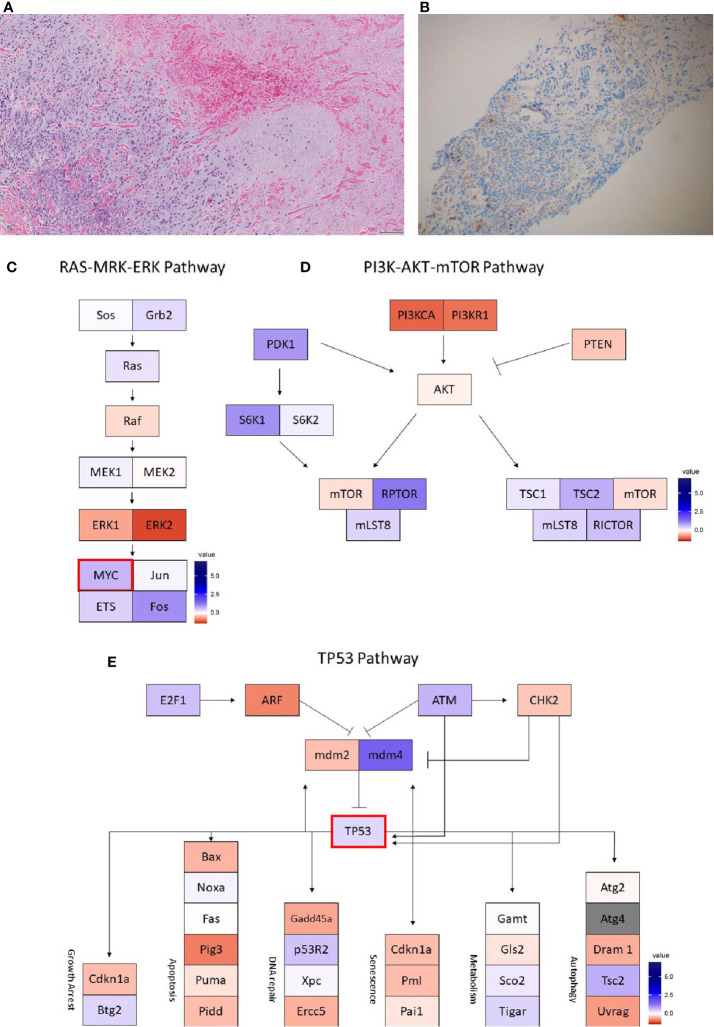
Case 5 **(A)** H&E image, showing metaplastic squamous carcinoma **(B)** PD-L1 by the Ventana PD-L1 SP263 assay. Images **(C–E)** show RNA expression heatmaps with modified z-scores of expression vs. non-metaplastic TNBC cases in pathways of interest for metaplastic breast cancer **(C)** RAS-MEK-ERK, **(D)** PI3K-AKT-mTOR **(E)** TP53. Genes with DNA mutations are outlined in red.

4 of the 5 patients were treated on a phase 1b trial evaluating the safety of paclitaxel or capecitabine in combination with the anti-PD-1 antibody, pembrolizumab. Inclusion criteria for this trial included ER/PR <1% by IHC, HER2 negative (IHC 0-1 or IHC2 with ISH HER2/CEP17 <2), measurable disease by RECISTv1.1, ECOG 0-1, and investigator-determined indication for paclitaxel or capecitabine in the 1^st^ or 2^nd^ line setting (17). One additional patient was treated with compassionate use nivolumab with bicalutamide and was not part of the trial. Because bicalutamide was discontinued shortly after commencing therapy, this case is still described in the series. Baseline biopsies prior to receiving anti-PD-1 therapy were available for all patients, as were post-treatment biopsies for Cases 1 and 3. All biopsies were reviewed by a pathologist to confirm the diagnosis of MBC (**Figures 1A**, **2A**, **3A**, **4A**, **5A**). All biopsies were also evaluated for PD-L1 expression in both tumor cells and immune cells with the Ventana PD-L1 SP263 assay and were reviewed by a pathologist for scoring (**Figures 1B**, **2B**, **3B**, **4B**, **5B**). A combined positive score (CPS), defined as the total number of PD-L1 staining cells (tumor cells, lymphocytes, and macrophages) divided by the total of viable tumor cells, multiplied by 100, is reported, with a CPS ≥ 1 considered positive per manufacturer insert, though recent trials in breast cancer have identified a higher cut-off of CPS ≥10 for clinical activity (18, 19). TILs were also scored by a pathologist per the International TILs Working Group guidelines for evaluating TILs in breast cancer (20).

### Biomarker Assessment

When tissue was available, additional exploratory biomarker immune profiling was conducted. Cases 2, 4, and 5 were evaluated with a multiplexed immunofluorescence (mIF) panel as part of the clinical trial of pembrolizumab + chemotherapy in which they were enrolled (17). These cases were compared to the non-metaplastic TNBC cases from the same clinical trial, also evaluated with mIF.

5μm Formalin Fixed Paraffin Embedded (FFPE) slides were stained and microwave treated in citrate buffer pH 6.0 to present cross-reactivity between antibodies. Tissue slides were incubated with DAPI as counterstain and coverslipped with VectaShield mounting media (Vector Labs). Whole slides were scanned and digitized at 10x magnification (PerkinElmer Vectra 3.0) for gross visualization of the tumor, with regions of interest scanned at 20x (0.36mm^2^) for quantification. The maximum possible number of non-overlapping regions of interest, as determined as areas with viable tumor and visible immune cells, were obtained for each slide. InForm software (PerkinElmer, package 2.4) was used according to manufacture instructions to segment and phenotype cells, with cells identified as cytokeratin-positive tumor cells, CD3-positive CD8-negative FoxP3-negative T-cells (Helper T-cells), CD3-positive CD8-positive T-cells (Cytotoxic T-cells), CD3-positive FoxP3-positive T-cells (Regulatory T-cells), and CD163-positive cells (Macrophages). PD-L1 quantitative immunofluorescence was also measured for each cell, which recent studies have found to be comparable to clinical PD-L1 scores (21, 22).

### Genomic Assessment

Cases were evaluated for targetable DNA mutations with a solid tumor mutation panel, although the commercial panels used varied as they were ordered at the discretion of the treating physician. All panels were processed similarly, with FFPE tissue sections examined by a pathologist and genomic DNA extracted from areas of viable tumor. Mutations were screened for by massively-parallel sequencing-by-synthesis.

RNA sequencing was performed on Cases 1, 2, 4, and 5 as part of exploratory analyses of the clinical trial. FFPE tissue sections were deparaffinized followed by RNA extraction and purification using the Qiagen AllPrep DNA/RNA FFPE kit. 85ng of input RNA was used to prepare sequencing libraries using the Illumina TruSeq RNA Exome kit. Sequencing of the RNA Exome libraries was performed on the Illumina HiSeq 4000 instrument at 2 x 76 read paired end configuration. Gene expression counts were quantified using salmon-v.0.11.2 (23). Differential gene expression analysis was performed using the R software package edgeR (24). Previously identified genes of interest in MBC were evaluated, including *AKT1, CCND3, CCNE1, CDK2NB, CDKN2A, CREB1, CREBBP, EGFR, KDM6A, KMT2D-MLL2, MKI67 (Ki-67), MTOR, MYC, Nanog, NF2, CD274 (PD-L1), PI3K, PIK3RI, PTEN*, and *TP53* (8, 9, 25, 26).

Peripheral blood T-cell receptor (TCR) sequencing was performed in cases 1, 2, 4, and 5, and on n=21 non-metaplastic metastatic TNBC patients from the phase Ib trial. Peripheral blood mononuclear cells (PMBCs) were collected at baseline and at regular intervals during treatment, and T-cell DNA was extracted and submitted for deep sequencing using the immunoSEQ Assay (Adaptive Biotechnologies). T-cell richness was estimated by the nonparametric model iChao1 function, and clonality index was calculated as the square root of the Simpson’s diversity index.

### Statistical Methods

For the purpose of hypothesis generation, immune and genomic profiles were constructed for individual patients using the above biomarkers data. For each biomarker outcome, raw scores were converted into modified z-scores, based upon underlying median and median absolute deviations of the outcomes across a cohort of TNBC patients treated on the aforementioned phase Ib chemo-immunotherapy clinical trial. Because of the limited sample size, this analysis was conducted primarily for hypothesis generation and to identify possible outlier features of the case tumors, which could potentially assist with characterizing the unique clinical response profiles of each case in the series.

## Results

### Case 1

The patient is a 63-year-old woman found to have a right breast mass on screening mammography, with biopsy showing a grade 3 invasive ductal carcinoma, ER-, PR-, HER2- (2+ IHC, ISH 3.04, ratio1.27). MRI additionally noted a small enhancing mass of the left breast, biopsy showing a concurrent grade 1 invasive ductal carcinoma with associated low-grade DCIS, ER >95%, PR 30%, HER2- (1+ IHC). She was treated with neoadjuvant therapy on the I-SPY trial with paclitaxel + ganetespib followed by doxorubicin + cyclophosphamide with a brief clinical response, followed by re-growth. She underwent bilateral mastectomy and sentinel lymph node biopsy, pathology consistent with metaplastic carcinoma, 3.1 cm x 2.8 cm with lymphovascular invasion, negative for perineural invasion, 2/2 intramammary lymph nodes involved with no extracapsular extension, 0/7 axillary nodes positive, 1/2 sentinel nodes with micro-metastatic carcinoma, no extracapsular extension, 0/11 additional axillary lymph nodes, and an RCB score of 3.835, class RCB-III (corresponding with suboptimal response and prognosis) (27). No residual carcinoma was detected on the left, 0/2 sentinel lymph nodes involved. She received adjuvant radiation therapy. However, follow up imaging noted an 8.3 cm right middle lobe perihilar mass with complete occlusion of the bronchus intermedius. Biopsy was obtained by bronchoscopy, with pathology showing a poorly differentiated malignant neoplasm consistent with metaplastic breast cancer, ER-, PR-, HER2-. She received palliative bronchoscopic debulking.

She was enrolled in a phase Ib trial, receiving pembrolizumab (200 mg IV every 3 weeks) with capecitabine (2000mg twice daily by mouth on days 1-7, every 2 weeks) (17). Per trial protocol, CT imaging of the chest, abdomen, and pelvis were obtained at baseline and every 12 weeks thereafter to assess for response by RECIST v1.1. Imaging at 12 weeks showed an overall partial response, though with mixed findings showing significant shrinkage of her dominant tumor, but enlargement of a left lung lesion (**Figures 1C–F**). The left lung lesion was biopsied and was consistent with metaplastic breast cancer. She received palliative radiation to her right lung mass. On follow up at 23 weeks, had developed new scalp lesions, which were biopsied and consistent with metaplastic breast cancer. She subsequently enrolled in hospice.

PD-L1 expression and TILs were evaluated by a pathologist on pre- treatment and post- treatment biopsies. PD-L1 expression on tumor cells was 0% on both pre- treatment and post- treatment biopsies, but 10% and 40% respectively on immune cells. CPS measured 5 on the pre-treatment biopsy and 1.5 on post-treatment biopsies, both above the threshold for positivity of ≥ 1, but below the ≥10% threshold. TILs were 20% in the pre-treatment biopsy but decreased to 1% in the post- treatment biopsy.

DNA mutations noted included *PIK3CA, TP53, PTEN, CDKN2A*. In a comparison of RNA expression, there were no marked differences in expression within the *TP53* or the *RAS/MRK/ERK* pathways, but *PDK1* appeared less expressed within the *PI3K* pathway compared to other cases (**Figures 1G–I**).

### Case 2

The patient is a 58-year-old woman who presented with a gradually enlarging right breast, biopsy revealing a grade 3 invasive ductal carcinoma, ER-, PR-, HER2- (IHC 0, FISH ratio 1.23). Right axillary lymph node biopsy was positive for metastatic breast carcinoma. She received neoadjuvant dose-dense doxorubicin + cyclophosphamide, followed by paclitaxel, with decrease in the right breast mass but increase in an axillary dominant node on follow up ultrasound. She underwent lumpectomy and axillary lymph node dissection, with pathology showing a grade 3 invasive ductal carcinoma, 4.0 cm, with an additional 8 mm focus, 3/19 lymph nodes positive with the largest at 2.4 cm, negative for lymphovascular invasion. She received adjuvant radiation to the right breast. She later presented for follow up and reported increasing mid-back pain, with MRI of the T- and L-spine without evidence of metastasis to the spine, but found enhancing pulmonary lesions. CT chest noted bilateral lung lesions, with core biopsy showing an ER-, PR-, HER2- breast cancer with metaplastic features with focal chondroid differentiation.

She enrolled in the aforementioned phase Ib trial of capecitabine + pembrolizumab. Follow up CT scans at 12 weeks showed a partial response, with an overall shrinking of multiple lung nodules, while also noting growth of other smaller nodules (**Figures 2C–E**). However, follow up scans at 24 weeks showed clear progression of disease and she was taken off the trial. She remains on 6^th^ line therapy with sacituzimab as of March 2021, with addition lines including eribulin, gemcitabine, cisplatin, and paclitaxel.

**Figure 6:**
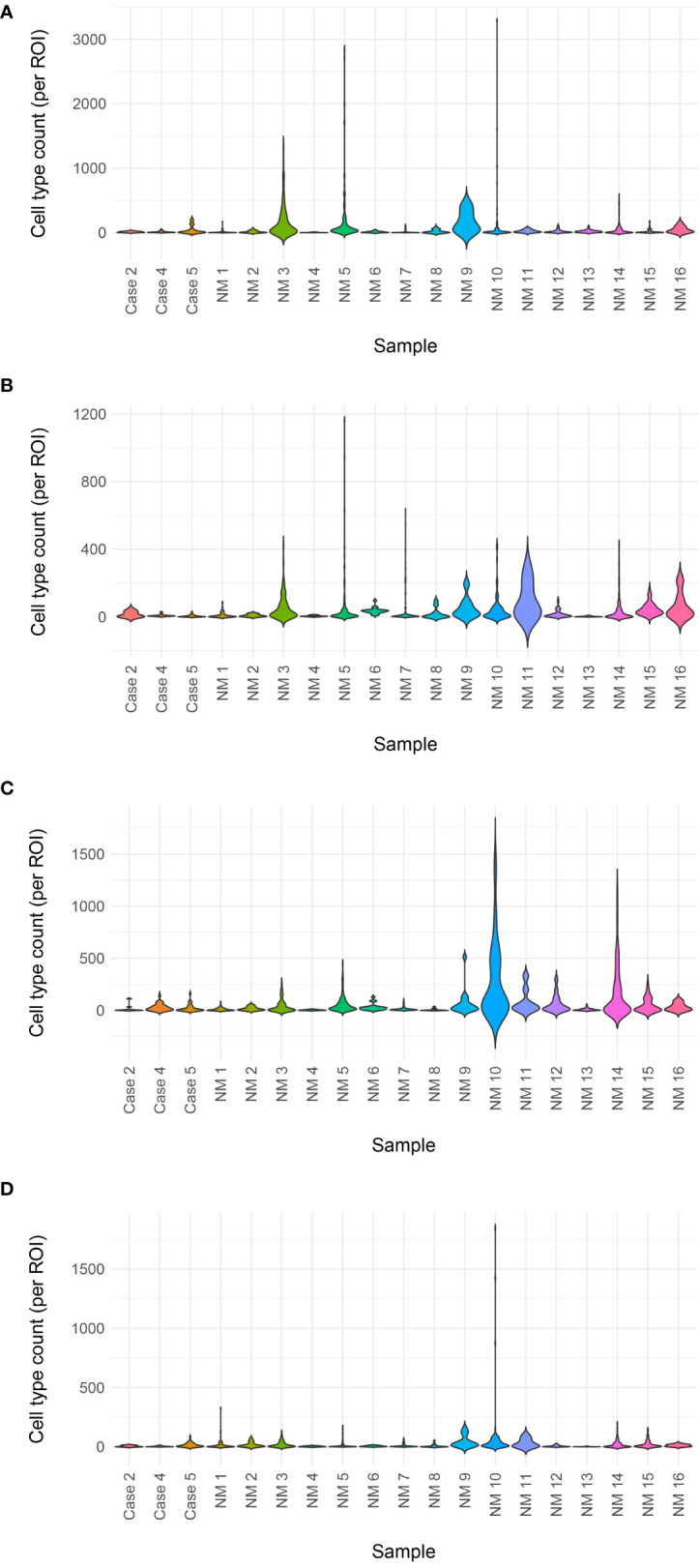
Immune cell counts by mIF. Total immune cell counts for metaplastic Cases 2, 4, and 5 plotted with non-metaplastic (cases identified as ‘NM’) TNBC from the same clinical trial in a violin plot. No clear difference is noted between the metaplastic cases and non-metaplastic cases **(A)** Helper T-cells **(B)** Cytotoxic T-cells **(C)** Macrophages **(D)** Regulatory T-cells.

On pre-treatment biopsy, PD-L1 expression was noted on 0% of tumor cells and 10% of immune cells, with a CPS of 5, above the threshold for positivity of ≥ 1, but below the ≥ 10 threshold. PD-L1 scoring by mIF was relatively low. TILs were scored as 15%. Immune cell counts were lower for CD8+ Cytotoxic T-cells, CD163+ Macrophages, and FOXP3+ Regulatory T-cells compared to non-metaplastic cases, but CD3+ Helper T-cells were higher than in non-metaplastics (**Table 1**, **Figure 6**). DNA mutations of interest included *TP53, MYC*, and *DICER1*. No significant patterns of increased or decreased expression was found in RNA analysis of the *TP53* or *PI3K* pathways. Higher expression was seen within the *RAS/MEK/ERK* pathway (**Figures 2F–H**).

**Table 1:** Immune cell counts in Case 2 by mIF.

Patient	Median raw cell count per ROI (CD3+)	Z-score *vs.* Non-metaplastic (CD3+)	Median raw cell count per ROI (CD8+)	Z-score *vs.* Non-metaplastic (CD8+)	Median raw cell count per ROI (CD163+)	Z-score *vs.* Non-metaplastic (CD163+)	Median raw cell count per ROI (FOXP3+)	Z-score vs. Non-metaplastic (FOXP3+)
**Case 2**	10.7	0.85	14.5	-0.51	16.9	-0.67	5.1	-0.82

ROI, region of interest; CD3+, CD3-positive CD8-negative FoxP3-negative T-cells (Helper T-cells); CD8+, CD3-positive CD8-positive T-cells (Cytotoxic T-cells); CD163+, CD163-positive cells (Macrophages); FOXP3+; CD3-positive FoxP3-positive T-cells (Regulatory T-cells).

### Case 3

The patient is an 82-year-old woman with a prior history of right sided stage IIB breast cancer in 2001, treated with mastectomy and axillary lymph node dissection, ER+, PR+, HER2-. She received adjuvant chemotherapy with cyclophosphamide, epirubicin, and 5-FU for 6 cycles, and additionally received radiation, 5 years of tamoxifen and 7 years of aromatase inhibitors (letrozole and exemestane). She had normal surveillance mammographies until November 2015 where she was found to have calcifications and possible distortion in the left upper outer breast. Biopsy found grade II pleomorphic invasive lobular carcinoma, ER 2%, PR-, HER2- (IHC 2+, ISH 1.8, ratio 1.06). She had a left breast mastectomy with sentinel lymph node biopsy, with a 5 mm residual invasive lobular carcinoma, with additional foci ranging from 1-3 mm, grade II, with negative margins, and extensive lymphovascular invasion, 2/2 sentinel nodes positive. She received adjuvant cyclophosphamide, methotrexate and 5-FU.

She developed a local chest wall recurrence, biopsy showing a metaplastic breast carcinoma with a component of pleomorphic lobular carcinoma associated with squamous differentiation, ER 20%, PR-, HER2- (IHC 1+ ISH 2.3, ratio 1.1), with androgen receptor staining positive in 30% of tumor cells. She received radiation, and then was started on fulvestrant + palbociclib, but had disease progression. She then started on exemestane + everolimus, but again had progressing skin lesions. She was then started on 3^rd^ line compassionate use nivolumab with off-label bicalutamide as the patient had wanted to avoid further chemotherapy, and had not previously responded to ER-directed therapy. Bicalutamide was held after 2 weeks of treatment, with concerns for fluid retention and swelling. At 1 month follow up she had worsening skin lesions, but nivolumab was continued with the possibility of a flare reaction causing the exam findings rather than disease progression. 2 months into treatment skin lesions appeared to be crusting over, and at 4 months appeared to have a complete response (**Figures 3C–E**). She continued on therapy for an additional 4 months when new skin lesions were noted on her back and trunk and a biopsy confirmed disease recurrence.

PD-L1 expression on tumor cells was 2% of pre-treatment and 0% of post-treatment tumor cells were positive for PD-L1, compared to 50% of both pre-treatment and post-treatment immune cells. CPS was above the threshold for positivity of ≥ 1, and a higher threshold of ≥ 10 in the pre-treatment sample with a CPS of 10, though only above the ≥ 1 threshold in the post-treatment sample with a CPS of 3. TILs were scored as 30% in the pre- treatment and 15% in the post-treatment samples. DNA mutations included *PIK3CA, TP53, AKT1, CDH1, KMT2D.* Further genomic and immunoprofiling was unavailable for this case, as this patient was not a part of the clinical trial.

### Case 4

The patient is a 60-year-old woman who presented with a painful large left breast mass. Biopsy of the left breast showed grade 3 invasive ductal carcinoma with focal spindle cell features, also noted on left axillary biopsy, ER-, PR-, HER2- (IHC 0, ISH 1.55, ratio 0.86). She received 4 cycles of neoadjuvant dose dense doxorubicin + cyclophosphamide with minimal response, followed by 4 cycles of carboplatin + weekly paclitaxel with some response. She underwent a left modified radical mastectomy, with pathology showing a 4.4 cm grade 3 IDC with metaplastic features, and extensive lymphovascular invasion, clear surgical margins, and 4/7 axillary lymph nodes involved with extranodal extension. Prior to receiving adjuvant radiation, a subcutaneous nodule was found inferior to her mastectomy incision, with excisional biopsy showing 3 foci of recurrent/residual IDC with sarcomatoid features, with one focus extending beyond the excisional margin. She received adjuvant radiation, and a subsequent PET scan and brain MRI were without evidence of residual disease. She then presented with left arm swelling, CT chest, abdomen, pelvis found enlarged lymph nodes in the neck and chest, multiple pulmonary nodules, small hypodensities in the liver measuring less than 5 mm, and sclerotic-appearing lesions in the manubrium. A brain MRI and bone scan showed no evidence of metastases. An ultrasound-guided FNA of a neck nodule on the right showed extensive necrosis and degenerated atypical cells, consistent with a necrotic carcinoma.

She was enrolled in the same phase Ib trial of capecitabine + pembrolizumab. Follow up imaging at 12 weeks noted a mixed response with growth of some nodes and regression of others, but she did have a new bony metastasis at T11 and was taken off of the trial.

On pre-treatment biopsy, PD-L1 expression was noted on 0% of tumor cells and 2% of immune cells, with a CPS of 0.5, under the threshold for positivity of ≥ 1. PD-L1 scoring by mIF was lower than the median of cases evaluated. TILs were scored as 2%. Immune cell counts by mIF noted higher CD163+ Macrophages than in non-metaplastic cases, and lower FOXP3+ Regulatory T-cells, which were 4^th^ lowest among the 19 evaluable cases. CD3+ Helper T-cells and CD8+ Cytotoxic T-cell counts were similar to non-metaplastic cases (**Table 2**, **Figure 6**). DNA mutations of interest included *PIK3R1, CHEK2, NF1*, and *NCOR1*. RNA expression in the *TP53* pathway found decreased *MDM4* and *CHK2*, but otherwise was without a clear pattern of increased or decreased expression. The *PI3K* pathway noted increased *PTEN*, but otherwise was again without a clear pattern through the rest of the pathway. Strong expression was seen in the *RAS/MRK/ERK* pathway, particularly of *JUN* and *FOS* (**Figures 4C–E**).

**Table 2:** Immune cell counts in Case 3 by mIF.

Patient	Median raw cell count per ROI (CD3+)	Z-score *vs.* Non-metaplastic (CD3+)	Median raw cell count per ROI (CD8+)	Z-score *vs.* Non-metaplastic (CD8+)	Median raw cell count per ROI (CD163+)	Z-score *vs.* Non-metaplastic (CD163+)	Median raw cell count per ROI (FOXP3+)	Z-score *vs.* Non-metaplastic (FOXP3+)
**Case 4**	5.5	0.04	5.5	-0.33	18.5	0.92	1.5	-0.67

ROI, region of interest; CD3+, CD3-positive CD8-negative FoxP3-negative T-cells (Helper T-cells); CD8+, CD3-positive CD8-positive T-cells (Cytotoxic T-cells;, CD163+, CD163-positive cells (Macrophages); FOXP3+, CD3-positive FoxP3-positive T-cells (Regulatory T-cells).

### Case 5

This is a 62-year-old woman who had a small left breast lump that rapidly grew into a fungating mass. Skin punch and core needle biopsies showed metaplastic carcinoma with extensive necrosis and dermal direct extension, ER-, PR-, HER2- (IHC 0, ISH 3.05, ratio 0.72). Staging CT scan revealed a large left breast mass measuring 13.6 cm with a large left axillary node measuring 7.4 cm, numerous bilateral pulmonary metastasis, a suspected metastatic pancreatic neck mass measuring 1.8 cm, and a soft tissue lesion surrounding the right 10^th^ rib, without other definite bone metastases, but bone scan noted multiple bone metastases.

She received paclitaxel (80mg/m2 IV weekly on days 1, 8, 15 of each 3-week cycle) with pembrolizumab (200 mg IV every 3 weeks). Following initiation, she had a mild infusion reaction to paclitaxel, but was maintained on therapy with dexamethasone pretreatment. The patient felt her breast mass shrank initially, but on follow up appointment prior to cycle 3, her mass appeared larger and repeat CT of the chest, abdomen, and pelvis showed progressive disease at multiple foci with a new pathologic fracture of the L-spine. She received palliative radiation to her spine and was taken off the trial and started on a DAE regimen (doxorubicin 30mg/m2 IV q3wk, bevacizumab 15mg/kg q3wk, and everolimus 5mg PO daily). She developed disease progression and subsequently enrolled in hospice.

A pre-treatment biopsy was available for review and PD-L1 expression was noted on 0% of tumor cells and 10% of immune cells, with a combined positive score of 2, above the threshold for positivity of ≥ 1, but below the threshold of >10. PD-L1 scoring by mIF noted relatively low expression. TILs were scored as 5%. Immune cells by mIF noted higher FOXP3+ Regulatory T-cells than the median of non-metaplastic cases, as well as compared to the other metaplastic cases, but overall populations were low for all cases. CD3+ Helper T-cell counts were near median values, with both CD8+ Cytotoxic T-cell and CD163+ Macrophages lower than non-metaplastic cases (**Table 3**, **Figure 6**). DNA mutations of interest included *TP53* and *MYC.* No significant patterns were noted in RNA expression in the *TP53* and *RAS/MEK/ERK* pathway, though ERK2 was significantly lower in the *RAS/MEK/ERK pathway*. In the *PI3K* pathway, *PIK3CA* and *PIK3R1* were relatively lower, but no pattern of reduced expression was noted in the rest of the pathway (**Figures 5C–E**).

**Table 3:** Immune cell counts in Case 5 by mIF.

Patient	Median raw cell count per ROI (CD3+)	Z-score *vs.* Non-metaplastic (CD3+)	Median raw cell count per ROI (CD8+)	Z-score *vs.* Non-metaplastic (CD8+)	Median raw cell count per ROI (CD163+)	Z-score *vs.* Non-metaplastic (CD163+)	Median raw cell count per ROI (FOXP3+)	Z-score *vs.* Non-metaplastic (FOXP3+)
**Case 5**	6.5	0.22	2	-0.65	4.5	-0.47	5.5	0.52

ROI, region of interest; CD3+, CD3-positive CD8-negative FoxP3-negative T-cells (Helper T-cells); CD8+, CD3-positive CD8-positive T-cells (Cytotoxic T-cells); CD163+, CD163-positive cells (Macrophages); FOXP3+, CD3-positive FoxP3-positive T-cells (Regulatory T-cells).

### Comparative Biomarker Assessment of Metaplastic *versus* Non-Metaplastic TNBCs

The small sample size in this series prohibited extensive characterization of MBC. However, because data in MBC are limited due to the rarity of this disease, it was of interest to conduct an informal, hypothesis-generating descriptive comparison of immunoprofiles using MBC versus non-MBC specimens from the aforementioned phase Ib trial.

### PD-L1

**Figure 7:**
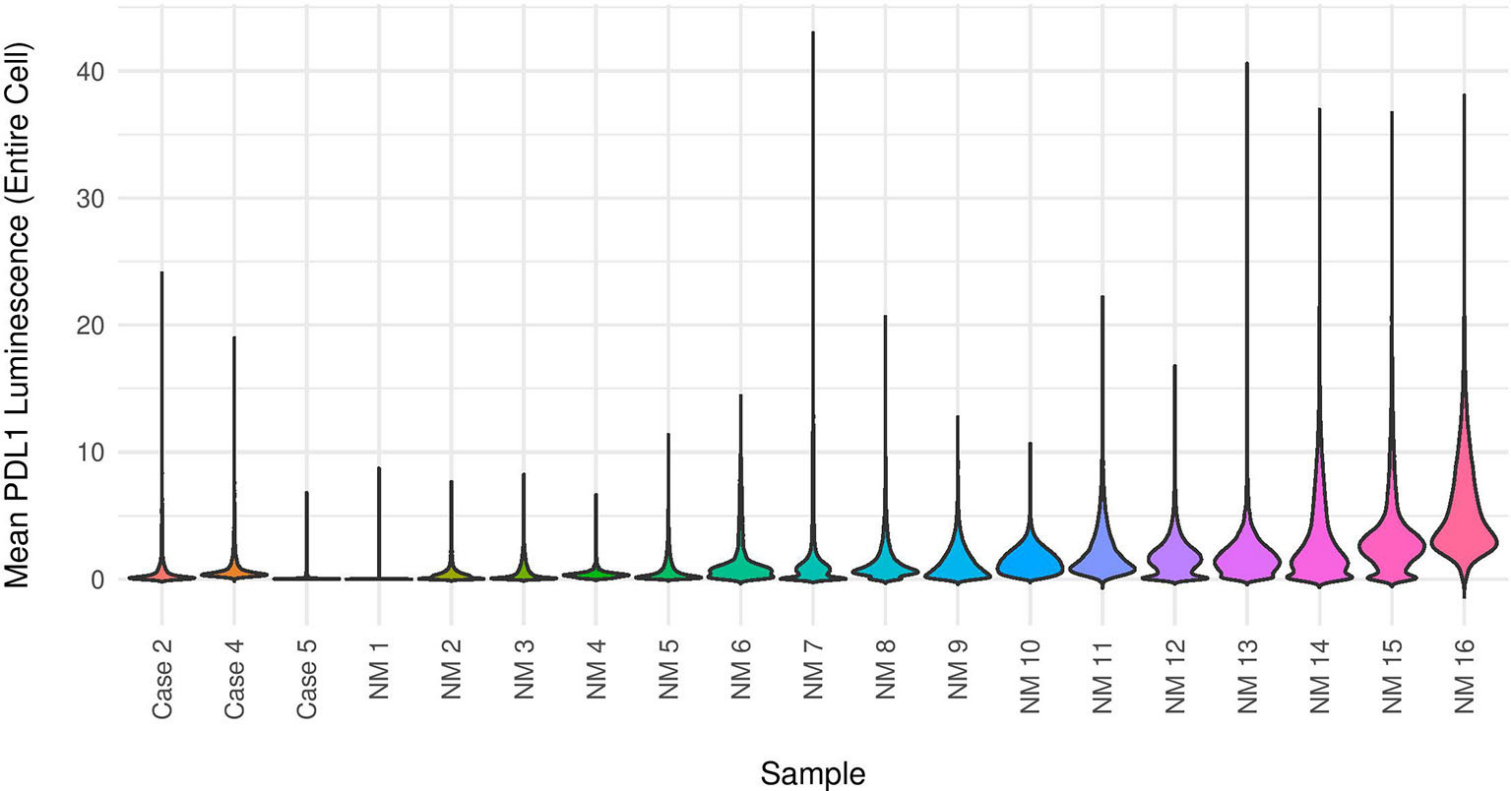
PD-L1 mIF violin plot. Mean PD-L1 quantitative immunofluorescence in baseline biopsies for metaplastic Cases 2, 4, and 5 as well as non-metaplastic (cases identified as ‘NM’) TNBC from the same clinical trial in a violin plot. A trend towards lower mean PD-L1 expression is noted in the metaplastic cases.

PD-L1 expression by mIF was generally lower in the metaplastic cases vs the non-metaplastic TNBCs, with all 3 cases evaluated below the median in PD-L1 expression (**Figure 7**). However, clinical PD-L1 scoring by CPS >1 showed that 4 of 5 metaplastic cases were positive by this definition with only Case 4 below this threshold. When using a higher cutoff of CPS ≥10 for positivity as in other recent trials of pembrolizumab in triple negative breast cancer, only Case 3 met the threshold (18).

### Immune Cells

Given the heterogeneity of MBC, comparisons were made between each metaplastic case and n=14 evaluable non-metaplastic TNBC cases to evaluate for outlier factors to differentiate metaplastic and non-metaplastic TNBC, rather than against all other cases including the 2 other metaplastic cases (n=16) in an attempt to identify the unique differences in each metaplastic case against TNBC, rather than a cohort that would include other metaplastic cases. Evaluation of immune cells by mIF demonstrated overall lower median raw cell counts across regions of interest in the metaplastic cases compared to the median of non-metaplastic TNBC cases. No obvious outliers were noted in comparison to non-metaplastic TNBC. Cases 2, 4, and 5 had positive z-scores in comparing CD3+ Helper T-cells to non-metaplastic TNBC, but all scores were <1 (**Table 4**). To additionally evaluate heterogeneity in MBC, the variance in immune cell counts between regions of interest was evaluated. A median absolute deviation was calculated and overall, less variance was seen in metaplastic cases compared to non-metaplastic cases (**Table 5**).

**Table 4:** Comparison of mIF cell counts.

Patient	Median raw cell count per ROI (CD3+)	Z-score *vs.* Non-metaplastic (CD3+)	Median raw cell count per ROI (CD8+)	Z-score *vs.* Non-metaplastic (CD8+)	Median raw cell count per ROI (CD163+)	Z-score *vs.* Non-metaplastic (CD163+)	Median raw cell count per ROI (FOXP3+)	Z-score *vs.* Non-metaplastic (FOXP3+)
**Case 2**	10	0.85	3.5	-0.51	2.5	-0.67	1	-0.82
**Case 4**	5.5	0.04	5.5	-0.33	18.5	0.92	1.5	-0.67
**Case 5**	6.5	0.22	2	-0.65	4.5	-0.47	5.5	0.52
**Non-metaplastic (n=16)**	5.25	n/a	9	n/a	9.25	n/a	3.75	n/a

ROI, region of interest; CD3+, CD3-positive CD8-negative FoxP3-negative T-cells (Helper T-cells); CD8+, CD3-positive CD8-positive T-cells (Cytotoxic T-cells); CD163+, CD163-positive cells (Macrophages); FOXP3+, CD3-positive FoxP3-positive T-cells (Regulatory T-cells).

Raw cell counts for immune cells as a median across regions of interest, quantified by a multiplexed immunofluorescence panel and calculated modified z-scores comparing metaplastic cases to n=16 non-metaplastic TNBC. Stromal and intra-tumor immune cells were not differentiated due to low numbers of immune cells within areas of tumor.

**Table 5:** Variance in immune cells by mIF reported as median absolute deviation.

Cell-type	Non-metaplastic (n=14)	Metaplastic (Cases 2, 4, 5)
CD3+	3.75	1
CD8+	7.25	1.5
CD163+	6.75	2
FOXP3+	2.25	0.5

CD3+, CD3-positive CD8-negative FoxP3-negative T-cells (Helper T-cells); CD8+, CD3-positive CD8-positive T-cells (Cytotoxic T-cells); CD163+; CD163-positive cells (Macrophages), FOXP3+; CD3-positive FoxP3-positive T-cells (Regulatory T-cells).

### RNA and TCR Sequencing

**Figure 8:**
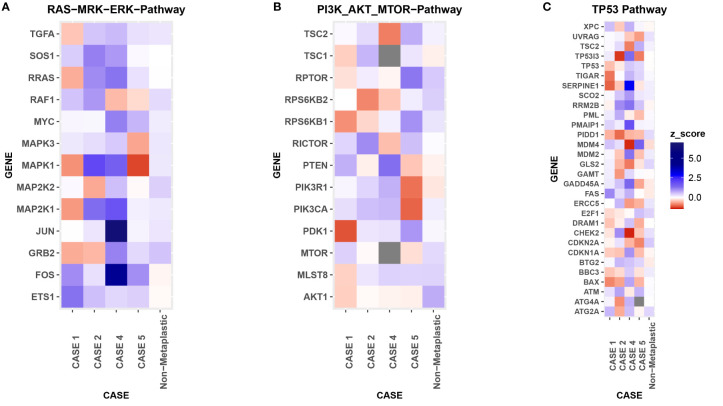
RNA Heat Map. RNA expression heatmaps with modified z-scores of expression vs non-metaplastics represented for each patient for 3 molecular pathways of interest in metaplastic breast cancer, **(A)** RAS-MRK-ERK pathway, **(B)** PI3K-AKT-mTOR pathway, **(C)** TP53 pathway.

Comparison of RNA sequencing did not demonstrate significant differences between metaplastic and non-metaplastic cases in multiple genes of interest, but did note multiple outlier genes, with an arbitrary cutoff of a modified z-score >3 in 2 or more metaplastic cases selected to identify possible outliers: *SOX8, CIC, COL9A3, ZFAND1, UBE2W, C2orf40, ENY2, RBM39, TGS1, DPY19L4, CLEC18A, ACAN, SLC25A32, VIRMA, IGF2, NOTUM, WWP2, NPIPB11, UPK1B, GABPB1, NR4A1, SLC25A42, FBXO25*. RNA expression in pathways of interest in MBC are further presented in **Figures 1**, **2**, **4**, **5** and **8**.

**Figure 9:**
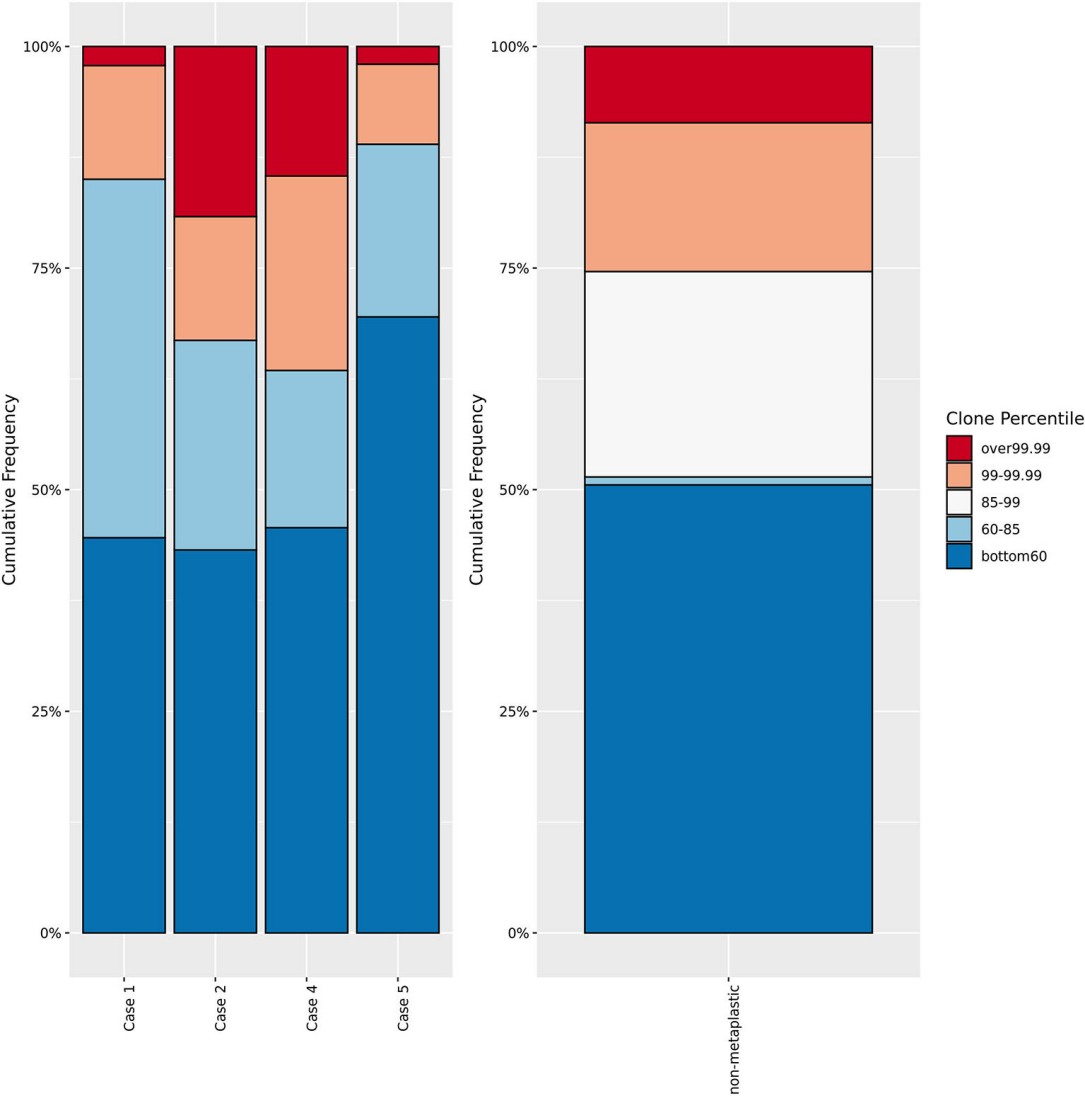
T-cell receptor sequencing clone frequency. Comparison of T-cell receptor sequencing clone frequency for metaplastic cases versus non-metaplastic TNBC prior to treatment. No significant difference in the percentage of low, low-middle, high-middle, or high frequency clones is noted in comparing the metaplastic versus non-metaplastic cases, with Cases 2 and 4 appearing to have more high frequency clones, and Cases 1 and 5 having less. Cases 1 and 2 were responders while Cases 4 and 5 did not respond to therapy.

TCR sequencing did not find significant changes in T-cell diversity by richness or clonality at baseline or during treatment between metaplastics and non-metaplastics. Evaluating the clonotype structure, metaplastics as a group vs non-metaplastics did not have significant differences in the amounts of higher frequency or lower frequency clones (**Figure 9**). However, Case 2 and Case 4 had a greater proportion of high-prevalence clones compared to other cases at baseline with Case 2 being a responder and Case 4 being a non-responder.

## Discussion

Our case series provides additional evidence of clinical activity of chemo-immunotherapy for MBC, a rare subtype of breast cancer for which limited outcomes data are available. In this series, we describe clinical responses in 2/4 cases treated with chemotherapy plus pembrolizumab. Of interest, we also report a fifth MBC case of a complete clinical response to nivolumab and bicalutamide. These data are supportive of previously published reports of clinical response in MBC. Adams reported a case of metastatic MBC with a large chest wall lesion that dramatically responded to nab-paclitaxel + pembrolizumab, with an ongoing response at 6 months (14), whereas Al Sayed et al. reported a case of chemo-refractory metastatic MBC treated with durvalumab + paclitaxel with a complete clinical response reported without recurrence at 2 years (15). In comparison, clinical response rates to chemo-immunotherapy among non-MBC TNBCs were 8/24 in the parent phase Ib clinical trial. Otherwise, a recent report of an MBC cohort within the DART trial (NCT02834013) of dual anti-CTLA-4 (ipilimumab) and anti-PD-1 (nivolumab) therapy reported responses in 3 of 17 patients (18%), with ongoing responses at 23, 25, and 27 months (16).

### Duration of Response and Mixed Responses

One notable observation from our series is that clinical responses were less durable than previously reported in published case reports, with progression free survival (PFS) of 5.3, 5.7 and 8.0 months for Cases 1, 2 and 3 respectively. Of note, the non-metaplastic TNBC responders (n=8) in the same trial as Cases 1 and 2 had an average PFS of 6.9 months, arguing that duration of response to chemo-immunotherapy in metaplastic breast cancer may not appreciably differ from non-metaplastic TNBC. However, notably, Case 3 which had the longest PFS was an ER+ tumor, treated with bicalutamide in addition to anti-PD-1 therapy, and not a TNBC, limiting direct comparisons.

A classic histologic trademark of MBC is intralesional heterogeneity, with the potential for having multiple regions of the tumor exhibiting distinct histologic features. In a recent analysis, it has also been suggested that intralesional histologic heterogeneity may reflect underlying genomic heterogeneity (28, 29). We evaluated for heterogeneity of radiographic response in our case series, and observed that Cases 1 and 2 had partial responses by RECIST v1.1, but had a mixed picture, with target lesions both shrinking and enlarging on initial follow up imaging. Case 4 also noted regression of some target lesions, but overall had disease progression by RECIST v1.1. Mixed responses, defined as the presence of simultaneously regressing and progressing target lesions, have been previously reported in studies with immunotherapy, with one study of stage IV melanoma treated with immune checkpoint blockade reporting 22% of patients with a mixed response. However, the majority of these cases do eventually become clear responders or progressors, and the phenomenon of a mixed response may be an artifact of the kinetics of immunotherapy, rather than being a separate outcome (30). In comparison, of 15 evaluable non-metaplastic TNBC, just 2 cases had similar mixed responses to chemo-immunotherapy (17). The limited sample size in this series prohibits drawing conclusions, however as additional MBC patients receive chemo-immunotherapy across the globe, it would be of interest to further evaluate the hypothesis that MBC could experience heterogeneous clinical responses. Because of the aggressive nature of this disease, and limited standard-of-care systemic options, it may be of value to consider locoregional therapy such as radiotherapy, to address progressive lesions in the setting of otherwise-responding disease. Notably, a recent study has shown promising activity and safety of radiotherapy + pembrolizumab in metastatic TNBC, with an objective response rate of 17.6% in a phase II trial of n=17 patients, although it is uncertain whether any of these were MBC. Another recent study in metastatic hormone receptor+/HER2- breast cancer did not show any responses with this combination in a heavily pre-treated group of n=8 patients (31, 32).

### PD-L1 Status and Response

Increased PD-L1 expression has been reported in multiple studies of MBC, with one study of 75 MBCs reporting PD-L1 overexpression in 46% of cases, with overexpression defined as 2+ staining in >5% of tumor cells, compared to just 9% in TNBC and 6% in HER2+ or ER/PR+ tumors (8). However, other studies have shown conflicting reports on rates of PD-L1 overexpression, potentially due in part to differences in how PD-L1 expression is measured and defined, with one study reporting 0% (0/18) expression (≥1% on tumor cells, SP142), and another reporting 50% (7/14) expression (>1% on immune cells and >+ by IHC, SP263) (25, 33). PD-L1 overexpression in MBCs may be related to epithelial to mesenchymal transition (EMT), which is thought to be related to the pathogenesis of MBC. MBC has been found to express markers of EMT including *ZEB1*, a repressor of E-cadherin and Yes-associated protein (34, 35). EMT may also explain the high rates of metastatic disease in MBC and has also been found to upregulate PD-L1 expression in breast cancer (36). Mutations of the *PI3K* pathway could also contribute to the overexpression of PD-L1 in MBCs (36, 37).

In this series, 4 of 5 cases exhibited modest PD-L1 expression, considered positive using the CPS overexpression by the CPS≥1 cutoff, but with only one case being positive by the ≥10 cutoff. In the phase III first-line KEYNOTE-355 trial, pembrolizumab was shown to improve outcomes in the CPS ≥10 group, but not the CPS≥1 group (18). In an exploratory analysis, this finding was also confirmed in the second/third-line trial of pembrolizumab versus chemotherapy, where an improvement in overall survival was noted in CPS ≥20, but not in CPS≥1 or CPS≥10 (19).In our series, 2 of the 3 MBC responders had a CPS of 1-10, with the 3^rd^ with a CPS of 10. These data raise the hypothesis that responses could be achieved in MBC even with modest PD-L1 expression levels. Because of the unmet need and absence of effective systemic options for MBC, further clinical investigation is warranted to determine whether the addition of anti-PD-1/L1 to chemotherapy would be effective for MBC cases with CPS 1-10.

### Genomic Profiling and PI3K Inhibition

Within previously identified genes of interest, 4 of 5 cases in our cohort had mutations of TP53, and 3 of 5 patients had mutations in the *PI3K* pathway, with Case 1 and Case 3 with *PIK3CA* mutations and Case 4 with a *PIK3R1* mutation (**Table 6**). This is particularly of interest in the context of immunotherapy as activating mutations of the *PI3K* pathway and loss of its antagonist *PTEN* have been found to have multiple effects on the tumor microenvironment. Loss of *PTEN* has been associated with increased expression of immunosuppressive cytokines, decreased tumor infiltration by T-cells, decreased T-cell mediated cell death, and increased PD-L1 expression (37, 38). Activating mutations of the *PI3K* pathway have been associated with resistance to PD-1/PD-L1 inhibition, by decreased expression of *IFN-γ* and granzyme B, and decreased CD8+ T-cell infiltration (39). Use of *PI3K* inhibitors has been found to result in decreased PD-L1 expression, increased CD8+ T-cells, and inhibition of regulatory T-cells, restoring the anti-tumor immune response (37, 40). Murine mammary models have suggested improved response to anti-PD-L1 therapy when used in combination with *PI3K* inhibitors (38, 41). Given the potential synergy of PI3K inhibition and immune therapy, a combination approach may warrant further investigation in this group of patients with high incidence of PI3K pathway alterations.

**Table 6:** Patient Characteristics.

Patient	WHO Subtype	ER/PR/HER2 Status	Clinically relevant mutations	PD-L1 (TC)	PD-L1 (IC)	CPS	TILs	Prior chemotherapy	Outcome
Case 1	Metaplastic squamous carcinoma	ER-/PR-/HER2-	PIK3CA, TP53, PTEN, CDKN2A	Pre: 0%	Pre: 10%	Pre: 5	Pre: 20%	Neoadjuvant: paclitaxel + ganetespib, doxorubicin + cyclophosphamide	Partial response, PFS: 5.3 months
				Post: 0%	Post: 40%	Post:1.5	Post: 1%		
Case 2	Metaplastic carcinoma with heterologous mesenchymal differentiation (chondroid)	ER-/PR-/HER2-	TP53, MYC, DICER1	0%	10%	5	15%	Neoadjuvant: doxorubicin + cyclophosphamide,paclitaxel	Partial response, PFS: 5.7 months
Case 3	Mixed metaplastic squamous carcinoma and pleomorphic invasive lobular carcinoma	ER+/PR-/HER2-	PIK3CA, TP53, AKT1, CDH1, KMT2D	Pre: 2%	Pre: 50%	Pre: 10	Pre: 30%	Adjuvant: Right breast: cyclophosphamide + epirubicin + 5-FU, tamoxifen, letrozole, exemestaneLeft breast: cyclophosphamide + methotrexate + 5-FUMetastatic 1st line: fulvestrant + palbociclibMetastatic 2nd line: exemestane + everolimus	Complete response, PFS: 8.0 months
				Post: 0%	Post:50%	Post:3	Post: 15%		
Case 4	Metaplastic squamous carcinoma	ER-/PR-/HER2-	PIK3R1, CHEK2, NF1, NCOR1	0%	2%	0.5	2%	Neoadjuvant: doxorubicin + cyclophosphamide, carboplatin + paclitaxel	Progressive disease
Case 5	Metaplastic carcinoma with heterologous mesenchymal differentiation (chondroid)	ER-/PR-/HER2-	TP53, MYC	0%	10%	2	5%	None	Progressive disease

ER, estrogen receptor; PR, progesterone receptor; HER2, human epidermal growth factor receptor 2; PD-L1, Programmed death-ligand 1; TC, tumor cells; IC, immune cells; CPS, combined positive score (# of PD-L1+ cells/total # of viable tumor cells x100), TIL (H&E): TIL scoring per the guidelines of the International TILs working group (20). PFS, progression free survival. Both pre- and post-treatment biopsies were available for Cases 1 and 3. Cases 2, 4, 5, only had pre-treatment biopsies available.

### Immunoprofiling of MBC

In addition to the above, in this series we also demonstrated a method of interrogating for unique immunologic and/or genomic features of individual tumor cases, relative to a parent cohort. While limited due to the small number of MBCs in this case series, we found no consistent or extreme differences in evaluation of immune cells, PD-L1 expression, RNA sequencing, or TCR sequencing in our MBC cases compared to non-metaplastic TNBC. Of note, a recent study evaluating 44 cases of MBC versus 174 cases of TNBC found more CD163+ cells in the stroma and less CD8+ cells in the tumor of MBC cases (44). This study also found higher PD-L1 expression in tumor cells of MBC (44). In contrast, MBC cases had low PD-L1 expression (**Figure 7**), with 4/5 cases as positive by a CPS ≥1, but only 1 of 5 positive with a threshold of CPS ≥10. RNA and TCR sequencing may additionally provide further insight into the biology of MBC, and while this series was too small to evaluate for distinguishing features of MBC, this framework of reporting Z-scores of cases relative to a parent cohort may be helpful in future case series of rare tumor types such as MBC. For example, evaluation of gene pathways of interest could help identify targeted treatments that may be more effective for individual cases of MBC given the heterogeneity of this disease process.

## Conclusion

Three patients demonstrated a response to therapy, albeit limited in duration. One responding patient exhibited low-level ER expression and pleomorphic lobular features, whereas the other cases were triple negative breast cancer. Responses were observed in tumors with intermediate PD-L1 expression (CPS 1-10). The aggressive nature of MBC and unmet need for effective palliative options, support further investigation of the role of anti-PD-1/L1 in PD-L1-intermediate MBC is warranted.

## Data Availability Statement

The original contributions presented in the study are included in the article/supplementary material, further inquiries can be directed to the corresponding author.

## Ethics Statement

The studies involving human participants were reviewed and approved by Providence Portland IRB and Cedars IRB. The patients/participants provided their written informed consent to participate in this study. Written informed consent was obtained from the individual(s) for the publication of any potentially identifiable images or data included in this article.

## Author Contributions

IK – data analysis, manuscript writing. VR – data analysis. BB – data analysis. BC – data analysis. YW – data analysis. MM – data analysis. ZS – data analysis. WR – data analysis. KS – data analysis. RB – treatment of study subjects. HM – treatment of study subjects. DP – treatment of study subjects, manuscript writing and review. All authors contributed to the article and approved the submitted version.

## Conflict of Interest

WR: Research support from Galectin Therapeutics, Bristol Myers Squibb (BMS), Merck, Tesaro/GlaxoSmithKline, MiNA Therapeutics, Inhibrx, Veana Therapeutics, Aeglea Biotherapeutics, Shimadzu, OncoSec, and Calibr. Patents/Royalties: Galectin Therapeutics. Advisory Boards: Nektar Therapeutics, Vesselon. DP: Received advisory board honoraria and institutional research funding support from Brooklyn ImmunoTherapeutics, Bristol-Myers Squibb, and Merck Laboratories. DP receives speaker bureau honoraria from Genentech and Novartis. Unrelated to this work, DP has received additional advisory board honoraria from other entities. RB: Consulting fees: Genomic Health, Astra Zeneca, Genentech; Institutional research funding: Seattle Genetics, Ichnos Biosciences, Merck; Honoraria for speakers bureau: Genentech, Seattle Genetics. HM: Consulting or Advisory Role - AstraZeneca; Bristol-Myers Squibb; Daiichi Sankyo; Genentech; Genomic Health; Immunomedics; Lilly; Merck; Pfizer; Puma Biotechnology; Puma Biotechnology; Seattle Genetics, Research Funding - Bristol-Myers Squibb (Inst); Lilly (Inst); Merck, (Inst); ZIOPHARM Oncology (Inst), Travel, Accommodations, Expenses - Amgen; AstraZeneca; Bristol-Myers Squibb; DAVA Pharmaceuticals; Genentech; Immunomedics; Lilly; Merck; Pfizer; Puma Biotechnology; Spectrum Pharmaceuticals, Other Relationship - Genomic Health; Lilly.

The remaining authors declare that the research was conducted in the absence of any commercial or financial relationships that could be construed as a potential conflict of interest.
